# The Editorial Management of This Journal

**Published:** 1883-07

**Authors:** 


### Editorial.

---

### THE EDITORIAL MANAGEMENT OF THIS JOURNAL.

When the names of the editors of this journal were, in January last, removed from its title-page and cover, it was announced that its editorial conduct would be hereafter strictly impersonal. This was an end to which, for some years past, the efforts of a small number of those interested in the success of the Medical Press Association, had been directed.

As a consequence, the last changes in the editorial staff were not published, and no such changes, should such occur, will be so announced hereafter. It has, however, appeared necessary to make at this time the statement that Dr. N. S. Davis, for some years associated with the management of this journal, retired from his connection with it last year, and, as a matter of fact, has not been responsible for any of its utterances since last December. The announcement of this fact is rendered necessary by the circumstance that many of our correspondents are still under the impression that Dr. Davis retains his connection with this journal, and are sending him communications and letters which should be addressed to *The Chicago Medical Press Association*, No. 188 South Clark Street.

In parting with the veteran journalist, clinician and physician, whose name has been for so many years associated in Chicago with its best medical activities of every kind, it need scarcely be said that his editorial *confrères*, bid him farewell with an unfeigned regret. Their intercourse, from the first day to the last, has been characterized by a mutual cordiality, and, on his part, by the utmost kindness and courtesy.

As he enters upon his new, and, we trust, broader field of journalistic labor, we extend to him our best wishes for his personal success and for the welfare of the periodical with which he is to be identified. If we shall ever be called upon to cross swords with him, in the generous warfare of public controversy on medical themes, we are sure that he will thrust and parry with no less fairness and conviction of the right, than that he has displayed from the first in the years during which he has been in the front of our ranks.

The anonymous editorial staff of this journal, in making this announcement, have thus no appeal to make for themselves either to the good will or the forbearance of its readers in particular, and the medical world in general. The fact is, that this journal stands no longer on the good will or forbearance extended to any one man or set of men. The JOURNAL stands on its own merits, and on these alone. If it is worthy of favor, it will, in the struggle where the fittest survive, accomplish its ends. If it cannot support itself in that struggle, it will go down; and what is more, it will deserve to go down. Just at present, it is not preparing for its latter end. Its bank balance is in the most satisfactory condition. It will be noticed that it is not frantically calling on its subscribers to pay up their arrears—a noteworthy symptom of dissolution in the weaker class of provincial papers, whether medical or political. As it publishes this four hundred and sixty-second number of its forty-sixth volume, conscious of the fact that it has survived its second dentition, and even passed its puberal epoch, it is not without a desire to prolong its days further, and to yet prove of usefulness in its day and generation. It is possessed of a keen sense of its possibilities in the future. It has been a sad and silent mourner at the funerals of not a few medical enterprises in journalism, whose earliest outgivings were rich in a promise of maturity. It has with greater delight watched the growth of several modest literary ventures in medicine, which now are its flourishing contemporaries. It has been the pleased or unwilling foster-parent of a nursery full of smaller sheets in this city and elsewhere.

On the whole, it has concluded to live for a while longer, and thus please its friends, if it has any, and disappoint its enemies, if there are such. The promise and potency of "matter" (in the typographical sense), is for it not without a confessed charm.

Know all men, therefore, to whom these presents shall come, that the CHICAGO MEDICAL JOURNAL AND EXAMINER proposes to continue business at the old stand, and respectfully solicits a continuance of the patronage which has actually begotten that sense of gratitude well defined as an ardent appreciation of favors to be bestowed in the future. "*Sint Maecenates, non deerunt, Flacce, Marones.*" (Martialis, Epigr. 3, viii., § 6.)

---

A MAN named Fulton, living among the Shuarkas in New Caladonia, left the tribe, and when two men were dispatched to get him back he shot both dead. The tribe being very mad about this affair they captured the man and—ate him; but every one of the Shuarkas who partook of the delicious meal died under symptoms of poisoning, the man having been syphilitic. The editor of the *Australasian Journal* is responsible for the truth of this story.

THE name of Professor DeLaskie Miller was accidentally left out of the Rush College advertisement in the June issue of the JOURNAL AND EXAMINER. The accident was not discovered until the entire edition had been mailed. He leaves the city in a few weeks for a trip to Europe.

Dr. W. T. BELFIELD has been appointed one of the attending surgeons of the Central Free Dispensary, in the department of genito-urinary diseases.

THE practitioners' courses in the several medical schools have been concluded, and those in attendance have for the most part left the city.

DR. H. P. MERRIMAN, of Chicago, has been elected Lecturer on Gynæcology in the spring course at Rush College.

The new pavilions of the Cook County Hospital are rapidly approaching completion.

